# Spirulina as a sustainable functional ingredient: nutrient density, bioactives, and food applications

**DOI:** 10.3389/fnut.2026.1810841

**Published:** 2026-04-15

**Authors:** Marwa Ezz El-Din Ibrahim, Selinay Demirel, Seydi Yıkmış, Nazan Tokatlı Demirok, Maisa Bushra Omer Ahmed, Amin K. Amin

**Affiliations:** 1 Department of Food and Nutrition Sciences, College of Agricultural and Food Sciences, King Faisal University, Al-Ahsa, Saudi Arabia; 2 Nutrition and Dietetics, Faculty of Health Sciences, Tekirdag Namik Kemal University, Tekirdağ, Türkiye; 3 Department of Food Technology, Tekirdag Namik Kemal University, Tekirdağ, Türkiye

**Keywords:** bioactive components, functional foods, microalgae, nutritional composition, sustainable nutrition

## Abstract

The growing demand for environmentally friendly, functional food sources underscores the importance of nutrient-rich alternatives. Spirulina (*Arthrospira platensis*), with its high protein content and rich micronutrient profile, has emerged as a promising candidate for this purpose. This review systematically evaluates the chemical composition, bioactive components, and potential health benefits relevant to food applications, drawing on peer-reviewed studies from 2015–2025. Spirulina biomass is characterized by a rich content of protein, complex carbohydrates, polyunsaturated fatty acids, vitamins, and minerals, while compounds such as C-phycocyanin, chlorophyll, β-carotene, and phenolics contribute significantly to antioxidant activity. Preclinical and limited clinical evidence indicate benefits, including reductions in oxidative stress and inflammation, improved glycemic control, improved lipid profile, and modulation of immune responses. Despite variations due to production conditions and potential contamination with heavy metals, Spirulina's low land and water requirements, along with high biomass yield, make it a sustainable protein source. Its incorporation into foods such as bakery and dairy products demonstrates potential for functional food development. Future research should concentrate on standardizing processing and encapsulation approaches to enhance sensory acceptance, and strategies such as taste masking, fermentation, and optimized product design will be key to achieving consumer-friendly, bioactive-rich products. This study emphasizes Spirulina's promise as a sustainable, functional food ingredient, and provides guidance for scalable application in the food industry.

## 1 Introduction

The rapid growth of the global population, environmental degradation, accelerated urbanization, and increasing economic pressures have intensified concerns regarding international food safety (1, 2). Specifically, although the world population is projected to reach nearly 9.8 billion by 2050, approximately 600 million people are still expected to experience hunger or malnutrition by 2030 (3, 4). This imbalance highlights the urgent need for sustainable, nutrient-dense, and economically accessible food sources. Traditionally, the basic protein requirement has been met from animal sources, and the recent interest in new protein-rich diet trends has distanced us from a sustainable and accessible nutrition model (1). Although plant proteins or grains are recommended as alternatives to animal sources for basic protein requirements, their bioavailability is lower than that of animal sources. In addition, limited arable land, the use of pesticides, and intensive agricultural practices negatively affect the expectation of productive crops in a vicious cycle (5). Therefore, identifying novel, sustainable protein sources that combine high nutritional quality with environmental resilience has become a priority for the food industry and public health sectors (6, 7). In this context, microalgae have considerable potential due to their valuable nutritional components, functional properties, diverse industrial applications, and health benefits (8).

Microalgae stand out as an alternative source of protein due to their high adaptability to environmental and ecological conditions, their eco-friendly and edible nature, and their therapeutic effects (9). *Arthrospira platensis* (*A. platensis*), also known as spirulina, has garnered attention in the food industry due to its high nutrient and protein content (10). Spirulina contains approximately 60%−70% protein by dry weight and is rich in essential amino acids, essential fatty acids, pigments, various bioactive compounds, vitamins, and minerals. In addition to its nutrient density, it is being touted as a “Superfood” for the future due to its potential antidiabetic, antioxidant, anti-inflammatory, immunomodulatory, and anticancer properties (11). These characteristics position Spirulina as both a functional food ingredient and a nutraceutical candidate. The commercial development of Spirulina has expanded significantly in recent years. It is currently used in a variety of industries, including the production of nutritional supplements, functional foods, drugs, personal care products, feed, and agricultural products (12). Spirulina is also used in the food industry to enhance its nutritional value and functional properties, being incorporated into bakery products, pasta, dairy formulations, snacks, confectionery, and beverages (6, 13).

Its cultivation advantages, such as growth in alkaline environments, reduced freshwater demand, and relatively low production costs, further strengthen its sustainability profile (14). Despite these promising attributes, several challenges limit the widespread application of Spirulina in food systems. Sensory drawbacks, particularly its characteristic fish-like odor, intense green color, and distinctive flavor, may negatively affect consumer acceptance (8, 9). Additionally, variations in cultivation conditions, harvesting techniques, processing methods, and drying technologies can influence its nutritional quality, bioavailability, safety profile, and economic feasibility (15, 16). Moreover, while many studies emphasize its health-promoting effects, inconsistencies remain regarding bioavailability, optimal dosage, technological compatibility in food matrices, and large-scale production sustainability (16, 17). Therefore, although Spirulina demonstrates strong potential as a sustainable and functional protein source, a comprehensive evaluation of its nutritional composition, technological performance, sensory implications, and industrial feasibility is required to support its broader integration into the food sector.

Accordingly, the aim of this review is to systematically and holistically examine the current scientific literature on Spirulina, focusing on its nutritional and biochemical characteristics, health-promoting effects, technological applications in food products, sustainability aspects, and existing limitations. By synthesizing recent evidence, this study seeks to clarify the current state of development, identify research gaps, and provide a comprehensive framework for future research and industrial implementation.

This review was compiled from major scientific databases, including PubMed, Web of Science, Scopus, and Google Scholar. The search was conducted using keywords such as “Spirulina,” “*Arthrospira platensis*,” “functional foods,” “bioactive compounds,” “health benefits,” and “sustainable nutrition,” with publications from 2015 to 2025. The study was based on peer-reviewed scientific articles in English that examined the nutritional and biochemical composition of Spirulina and its potential applications in food and health. Non-scientific studies lacking relevant nutritional or biochemical data were excluded. Additional references were identified by examining the citation chains of primary and current studies.

## 2 Nutritional value and chemical composition of Spirulina

Spirulina's nutritional and chemical properties can vary depending on growth conditions, geographical region, cultivation area, algal species, and environmental factors (18). Besides its rich protein concentration, Spirulina also includes approximately 15%−25% carbohydrates and 5%−8% lipids (19). Furthermore, this microalgal species is widely recognized for its antioxidant, probiotic, and therapeutic potential. Its rich composition of essential amino acids, polyunsaturated fatty acids (PUFAs), vitamins, minerals, and various bioactive compounds, combined with its high digestibility, makes it a precious content for food products (2, 6). The results of studies on macronutrient distribution during Spirulina production under different conditions are shown in Table 1.

**Table 1:** Macronutrient distribution of Spirulina produced under different conditions.

Approximate composition of dry weight (%- g/100 g)						
	**Protein**	**Carbohydrate**	**Lipid**	**Ash**	**Total dietary fiber**	**Moisture**	**References**
Macro-micro nutrients	63.9 ± 1.00	12.80 ± 0.21	10.70 ± 0.56	6.10 ± 0.10	5.80	7.90 ± 0.20	(28)
	65.71 ± 1.00	21.87 ± 0.44	6.94 ± 0.55	8.34 ± 0.06	-	5.81 ± 0.17	(133)
	61.57 ± 1.61	16.21	7.19 ± 0.25	7.10 ± 0.09	7.93 ± 0.38	5.37 ± 0.24	(24)
	64.18 ± 0.96	9.08 ± 0.06	4.53 ± 0.06	6.22 ± 0.09	8.86 ± 0.16	7.13 ± 0.1	(134)
	66.17 ± 0.99	7.94 ± 0.05	5.78 ± 0.08	6.34 ± 0.09	7.87 ± 0.15	5.90 ± 0.15	
	62.49 ± 0.94	9.25 ± 0.06	3.14 ± 0.04	10.47 ± 0.16	5.92 ± 0.11	8.73 ± 0.22	
	71.34 ± 0.21	6.83 ± 0.01	0.36 ± 0.01	5.93 ± 0.30	8.45 ± 0.19	7.09 ± 0.04	(135)
	47.04 ± 0.15	1.73 ± 0.36	36.39 ± 0.00	7.68 ± 0.04	-	7.16 ± 0.25	(32)
	80.00 ± 1.49	0.69	7.63 ± 1.87	11.67 ± 0.72	-	-	(20)
	54.60	22.23	6.12	8.28	-	8.77	(136)
	62.81	15.73	6.39	10.87	-	4.20	
	65.37 ± 0.05	-	0.20	10.73 ± 0.01	0.04	-	(137)
	57.75 ± 0.10	21.63 ± 0.46	6.81 ± 0.40	-	-	-	(21)
	48.73 ± 0.81	22.14 ± 0.11	7.32 ± 0.61	-	-	-	
	47.17 ± 0.02	18.22 ± 0.15	8.70 ± 0.25	-	-	-	
	41.16 ± 0.06	17.47 ± 0.18	8.70 ± 0.25	-	-	-	
	38.94 ± 0.29	16.55 ± 0.38	10.35 ± 0.55	-	-	-	
	53.12 ± 0.12	33.6 ± 1.90	2.87 ± 0.16	9.86 ± 0.35	-	-	(138)
	45.50 ± 2.33	21.00 ± 1.20	17.00 ± 1.10	9.33 ± 0.80	-	7.13 ± 0.54	(139)
	65.60 ± 2.50	19.40 ± 1.13	7.20 ± 0.62	6.50 ± 0.41	-	1.10 ± 0.10	(140)
	57.47	23.90	5.38	-	-	-	(141)
	64.15 ± 2.15	14.11 ± 2.70	6.61 ± 0.11	7.00 ± 0.20	7.80 ± 0.30	5.00 ± 1.00	(142)

SD, Standard Deviation.

The data presented in Table 1 are compiled from different published studies. Mean ± standard deviation (SD) values are shown where reported by the original authors. For studies that did not provide SD data, only mean values are included.

### 2.1 Macronutrient distribution

The macronutrient composition of Spirulina varies considerably across studies (Table 1). The high protein content reported by 26 results from optimizing the culture medium composition and cultivation parameters to maximize protein synthesis under laboratory conditions (20). Conversely, the variation in protein content reported by Hong et al. (21) may be attributed to Spirulina's ability to adapt its biochemical and physiological functions under environmental stress conditions, including elevated salinity and limited nutrient availability. Exposure to such stressors induces metabolic adjustments in the species to maintain cellular homeostasis and viability, thereby influencing its macronutrient profile (21). On the other hand, lipid content varies with physiological state and analytical methods; environmental stress conditions can affect lipid metabolism, leading to fluctuations in the proportion of this component (22). In conclusion, the observed variability in reported values may result from a range of biological and environmental factors, such as differences in culture medium composition, light exposure, nitrogen availability, and drying methods.

This compositional variability, especially given the factors to which proteins are exposed during processing, makes post-harvest preservation technologies critically important. In this context, microencapsulation stands out as an effective strategy for preserving the structural integrity of Spirulina proteins. This process shows that the essential components of the protein structure are largely preserved, and coating materials such as maltodextrin interact with the proteins, thereby maintaining functional properties. Although the high temperatures and mechanical forces applied during spray drying can cause partial protein degradation, the coating material mitigates this effect by forming a protective layer. Furthermore, Spirulina's high digestibility is preserved after microencapsulation; the system reduces exposure to oxygen and moisture, providing more stable release and a longer shelf life, especially in aqueous foods. Therefore, microencapsulation is an important technological solution contributing to the more stable and safe use of Spirulina in functional foods (23).

#### 2.1.1 Proteins and amino acids

Spirulina's main component is protein, accounting for 60%−70% of its dry weight (11). This microalga, distinguished by its elevated protein content, is a rich dietary source of vital amino acids, including isoleucine (7.9 ± 0.60%), leucine (7.9 ± 0.60%), lysine (2.70 ± 0.30%), methionine (2.50 ± 0.20%), phenylalanine (3.90 ± 0.30%), threonine (5.30 ± 0.30%), tryptophan, and valine (3.60 ± 0.30%), which collectively account for approximately 38.81% of the total protein fraction. In addition, it contains considerable levels of non-essential amino acids, such as alanine (2.90 ± 0.20%), arginine (4.80 ± 0.40%), glycine (1.40 ± 0.10%), glutamine (20.10 ± 0.80%), histidine, serine (5.10 ± 0.30%), and tyrosine (12.8 ± 2.40%) (20, 24). Furthermore, C-phycocyanin, a bioactive compound representing approximately 47% of the total protein content, plays a significant role in promoting human health due to its antioxidant and anti-inflammatory properties (13, 25). Additionally, due to its biological activities, C-phycocyanin has gained importance as a multipurpose ingredient across sectors such as personal care products, pharmaceuticals, agriculture, and animal nutrition. Spirulina, defined as a “Super Food” with these features, is used in place of other commonly consumed plant and animal protein sources due to its striking nutritional profile and high protein content (2, 26, 27).

#### 2.1.2 Carbohydrates

Spirulina contains carbohydrates as the most abundant component after protein content (19). On the other hand, changes in carbohydrate ratios during the production process, depending on conditions and cultivation methods, are shown in Table 1. An important structural feature of Spirulina is its thin peptidoglycan layer, characteristic of Gram-negative cyanobacteria, and its lack of a rigid, cellulose-containing cell wall. This structural feature allows for the easy digestion of carbohydrates in the human gastrointestinal system (28). The carbohydrate profile of Spirulina consists mainly of polysaccharides, including galactose, xylose, and arabinose, as well as monosaccharides such as glucose, rhamnose, and mannose. In addition, it contains trace amounts of galacturonic acid, glucosamine, ribose, and fructose (29).

The polysaccharides present in spirulina, together with other bioactive compounds, may help to prevent chronic disorders such as diabetes thanks to their antiviral, anticancer, anti-inflammatory, antioxidant, and immunomodulatory properties (17). Conversely, Spirulina-derived polysaccharides can be metabolized by the intestinal microbiota into butyrate, short-chain fatty acids (SCFAs), and other readily absorbable metabolites. This process promotes overall health by regulating the balance of intestinal microbiota and affecting the production of microbial metabolites (30). These properties make Spirulina an essential ingredient in functional foods and medical nutrition.

#### 2.1.3 Lipids and fatty acids

Although Spirulina has a low total fat content of 5%−8%, it stands out as a microalgae rich in unsaturated fatty acids with important biological activities for human health. Mainly containing PUFA such as gamma-linolenic acid (GLA), omega-3, and omega-6, it is nutritionally valuable (19, 31). The lipid profile of Spirulina includes palmitic acid, stearic acid, and arachidonic acid among saturated fatty acids, and oleic acid (n-9) among monounsaturated fatty acids. Spirulina's lipid profile includes stearic acid, arachidonic acid, and palmitic acid among saturated fatty acids. It also contains high levels of alpha-linoleic acid (ALA), eicosapentaenoic acid (EPA), and docosahexaenoic acid (DHA) (32, 33). Recent studies have shown that the quantitative composition of fatty acids varies with the growth conditions of Spirulina species. The qualitative and quantitative compositions examined in the studies are shown in Table 2.

**Table 2:** Fatty acid composition of Spirulina produced under different conditions.

Fatty acid composition of Spirulina in dry weight (g/100 g)						
**Fatty acids**	**(** **28** **)**	**(** **135** **)**	**(** **32** **)**	**(** **20** **)**	**(** **139** **)**	**(** **140** **)**
Palmitic Acid C16:0	2.56 ± 0.02	51.54 ± 2.26	37.09 ± 1.28	57.0. ± 4.60	-	42.85 ± 2.30
Palmitoleic Acid C16:1	0.29 ± 0.03	2.88 ± 1.57	-	2.20 ± 0.20	-	3.16 ± 0.10
Stearic Acid C18:0	0.12 ± 0.01	1.06 ± 0.28	5.50 ± 0.14	2.10 ± 0.40	-	1.00 ± 0.11
Oleic Acid C18:1 *cis9*	0.18 ± 0.02	2.69 ± 0.26	2.34 ± 0.11	6.00 ± 0.70	0.17 ± 0.007	3.53 ± 0.21
Linoleic Acid C18:2	1.15 ± 0.02	1.30 ± 0.04	3.33 ± 0.14	16.00 ± 0.70	0.36 ± 0.008	14.43 ± 1.10
Alpha-linoleic Acid C18:3	0.01 ± 0.01	-	4.28 ± 0.18	1.30 ± 0.10	-	0.47 ± 0.02
Gamma-linoleic Acid C18:3 cis6	1.66 ± 0.01	19.30 ± 0.59	24.45 ± 1.20	13.80 ± 0.40	22.76 ± 1.33	22.45 ± 2.10
Arachidic C20:0	-	-	6.24 ± 0.28	-	-	-
Total saturated fatty acids	2.70 ± 0.04	56.63 ± 0.89	48.83	59.10	23.65 ± 1.85	50.10 ± 2.20
Total unsaturated fatty acids	-	-	-	40.90	75.76 ± 5.40	49.20 ± 2.80
Total PUFA	0.01 ± 0.01	37.80 ± 0.76	46.02	32.70	71.63 ± 4.00	40.60 ± 2.30
Total MUFA	0.50 ± 0.05	5.57 ± 0.92	5.14	8.20	8.98 ± 0.55	8.50 ± 0.61

C16:0, Palmitic acid; C16:1, Palmitoleic acid; C18:0, Stearic acid; C18:1 cis 9, Oleic acid; C18:2, Linoleic acid; C18:3, Alpha-linoleic acid; C18:3 cis6, Gamma-linoleic acid; C20:0, Arachidic acid; MUFA, Monounsaturated fatty acids; PUFA, Polyunsaturated fatty acids; SD, Standard Deviation.

The data presented in Table 2 are compiled from different published studies. Mean ± standard deviation (SD) values are shown where reported by the original authors. For studies that did not provide SD data, only mean values are included.

#### 2.1.4 Vitamins and minerals

Vitamins are vital micronutrients that the human body cannot synthesize in adequate amounts and therefore must be obtained through dietary intake to support normal biochemical functions. Spirulina contains both water-soluble B vitamins and vitamin C, as well as fat-soluble vitamins E, A, and K. It contains high amounts of B group vitamins, especially thiamine (B1), riboflavin (B2), niacin (B3), pantothenic acid (B5), pyridoxine (B6), folic acid (B9), and cobalamin (B12) (24). It is thought to be helpful for vegan and vegetarian individuals who do not prefer animal sources in their diets, thanks to the B group vitamins it contains, in preventing vitamin B12 deficiency (2).

Spirulina is also a highly nutritious food, containing macrominerals and microminerals include potassium (K), selenium (Se), iron (Fe), magnesium (Mg), chromium (Cr), zinc (Zn), calcium (Ca), phosphorus (P), manganese (Mn), sodium (Na), boron (B), and copper (Cu) which are necessary for basic physiological functions (34). This rich vitamin and mineral content makes Spirulina suitable for use as a nutritional supplement and encourages its addition to different products as a functional food (2). Table 3 shows the vitamin and mineral composition of Spirulina, as revealed by various studies.

**Table 3:** Vitamin and mineral composition of Spirulina.

	References			
**Vitamins (mg/100 g)**	**(****24** **)**	**(** **134** **)**	**(** **134** **)**	**(** **134** **)**
Vitamin B1 (Thiamine)	5.61	0.63 ± 0.02	0.75 ± 0.02	1.08 ± 0.03
Vitamin B2 (Riboflavin)	4.94	2.12 ± 0.06	2.23 ± 0.07	3.84 ± 0.12
Vitamin B3 (Niacin)	17.19	8.21 ± 0.25	9.12 ± 0.27	12.83 ± 0.38
Vitamin B6 (Pyridoxine)	0.87	0.65 ± 0.20	0.68 ± 0.02	0.87 ± 0.02
Vitamin B12 (Cobalamin)	0.20	0.11 ± 0.00	0.11 ± 0.00	0.13 ± 0.00
Folic Acid	9.66	-	-	-
Vitamin A (Beta- Carotene)	243	185.21 ± 4.26	201.64 ± 4.64	325.45 ± 7.48
Vitamin E	9.57	-	-	-
Vitamin C	-	55.45 ± 1.66	61.84 ± 1.85	256.26 ± 6.41
Vitamin K	1.124	-	-	-
**Minerals (mg/100 g)**	**References**				
	**(** **134** **)**	**(** **24** **)**	**(** **34** **)**	**(** **32** **)**	**(** **143** **)**
Calcium (Ca)	850–1,185.57	1,043. 63	80	207.51 ± 3.54	855.466
Potassium(K)	1,385.37–1,678.72	2,185.74	62	1,675.00 ± 28.65	1.4274
Magnesium (Mg)	205.34–248.93	1.47	654.8	486.41 ± 20.51	372.63
Phosphorus (P)	-	1,917.71	-	728.22 ± 21.37	1.515
Sodium (Na)	85.77–120.45	1,510.16	45.2	2,033.33 ± 35.36	1.344
Chromium (Cr)	-	0.40	1.12	-	-
Iron (Fe)	21.32–46.58	338.76	125	16.27 ± 0.64	66.48
Copper (Cu)	-	1.50	0.321	-	-
Selenium (Se)	-	0.04	-	0.08 ± 0.00	0.031
Zinc (Zn)	1.23–1.85	4.49	8.26	0.19 ± 0.00	4.596
Boron (B)	-	3.56	-	-	-
Manganese (Mn)	-	7.01	5.23	1.09 ± 0.05	-

Thiamine, B1; Riboflavin, B2; Niacin, B3; Pantothenic acid, B5; Pyridoxine, B6; Folic acid, B9; Cobalamin, B12; Magnesium, Mg; Phosphorus, P; Potassium, K; Selenium, Se; Chromium, Cr; Calcium, Ca; Sodium, Na; Iron, Fe; Copper, Cu; Zinc, Zn; Boron, B; Manganese, Mn; SD, Standard Deviation.

The data presented in Table 3 are compiled from different published studies. Mean ± standard deviation (SD) values are shown where reported by the original authors. For studies that did not provide SD data, only mean values are included.

#### 2.1.5 Dietary fiber content

In recent years, dietary fiber has gained significant attention as an essential component of healthy nutrition and an important focus of health research (35). Some types of fiber are metabolized by gut bacteria, resulting in the formation of bioactive metabolites such as short-chain fatty acids. This process helps maintain microbiota balance, reduces the risk of constipation, reduces the frequency of gastrointestinal discomfort, alleviates inflammatory responses, and supports toxin elimination, providing various physiological benefits (35, 36). Through this mechanism, metabolites produced from dietary fiber modulate the immune system by influencing immune cell signaling pathways, helping to control excessive immune activation and lowering the risk of conditions such as diabetes, hypertension, and cardiovascular diseases (37).

A comprehensive understanding of the characteristics of dietary fiber is important for advancing the food industry and supporting the development of novel functional foods and nutritional supplements for the prevention and management of chronic diseases (38). In this regard, Spirulina has gained considerable interest due to its promising potential in the formulation of novel functional food products (39). The dietary fiber composition of Spirulina is influenced by several factors, including cultivation conditions, culture medium formulation, and drying techniques. According to data reported in the literature, the total dietary fiber content of Spirulina spans a wide range, reflecting the species' high adaptability to environmental conditions (Table 1) (9). These fiber fractions of Spirulina specifically support intestinal health and show potential for prebiotic effects (40).

Research reveals significant differences in the macronutrient and micronutrient content of Spirulina. This may be due to various factors such as drying methods, culture media, light intensity, and nitrogen levels. Because most studies are conducted under laboratory conditions, the impact of industrial production conditions on nutritional composition remains unclear. Future studies should focus on optimizing the nutritional value of Spirulina under standard environmental conditions.

#### 2.1.6 Pigments and phenolic compounds

Spirulina is a microalgae that stands out for its rich pigment content, particularly the C-phycocyanin pigment-protein complex that gives it its blue-green color (26). The stability of C-phycocyanin is primarily determined by temperature, pH, and osmotic stress. High temperatures, especially above 70–75 °C, irreversibly damage the protein structure, while pH deviations from the neutral range weaken intramolecular bonds. The presence of low levels of salt (NaCl) coats the pigment surface, partially stabilizing the protein structure and providing osmotic equilibrium. In food applications, this results in color loss, pigment degradation during processing, and a shorter shelf life. Therefore, the industrial use of C-phycocyanin is limited in high-temperature processes such as pasteurization and baking, and controlled pH and low-temperature strategies are required for effective use (41). In additionally, trace amounts of chlorophyll, β-carotene, phycobiliproteins, and other carotenoids are present (42, 43). The antioxidant potential observed in different extraction protocols is predominantly attributed to the bioactive pigment fractions, particularly C-phycocyanin and chlorophyll-a (44, 45). In addition, Bougatef et al. (46) reported in their study that phycocyanin extract not only exhibited the highest antioxidant potential among the tested fractions but also demonstrated remarkable antibacterial and antifungal properties, as well as significant cytotoxic effects against breast cancer cell lines (46).

On the other hand, Spirulina's nutritional value extends beyond its pigment content. It is also a microalgae rich in phenolic compounds. Although research has shown that Spirulina contains important bioactive compounds (e.g., vanillin, gallic acid, p-coumaric acid, chlorogenic acid, and caffeic acid), flavonoids (e.g., naringin and hesperidin), and other compounds (e.g., benzophenone, dinitrobenzoate, and quercetin), the type and amount of these compounds found in Spirulina are also affected by climate, production, and processing conditions (47, 48). Spirulina's phenolic compounds have been reported to exhibit significant biological activity against foodborne pathogens via methanol extraction. Consequently, this process supports food preservation while maintaining nutritional and sensory quality, thus extending the product's shelf life (49). Studies show that spirulina's rich pigment and phenolic profile are recognized as both natural coloring agents and health-supporting components with powerful oxidative, inflammation-reducing, viral-inhibiting, diabetes-reversing, and cholesterol-lowering effects (17).

## 3 Spirulina and health benefits

**Figure 1:**
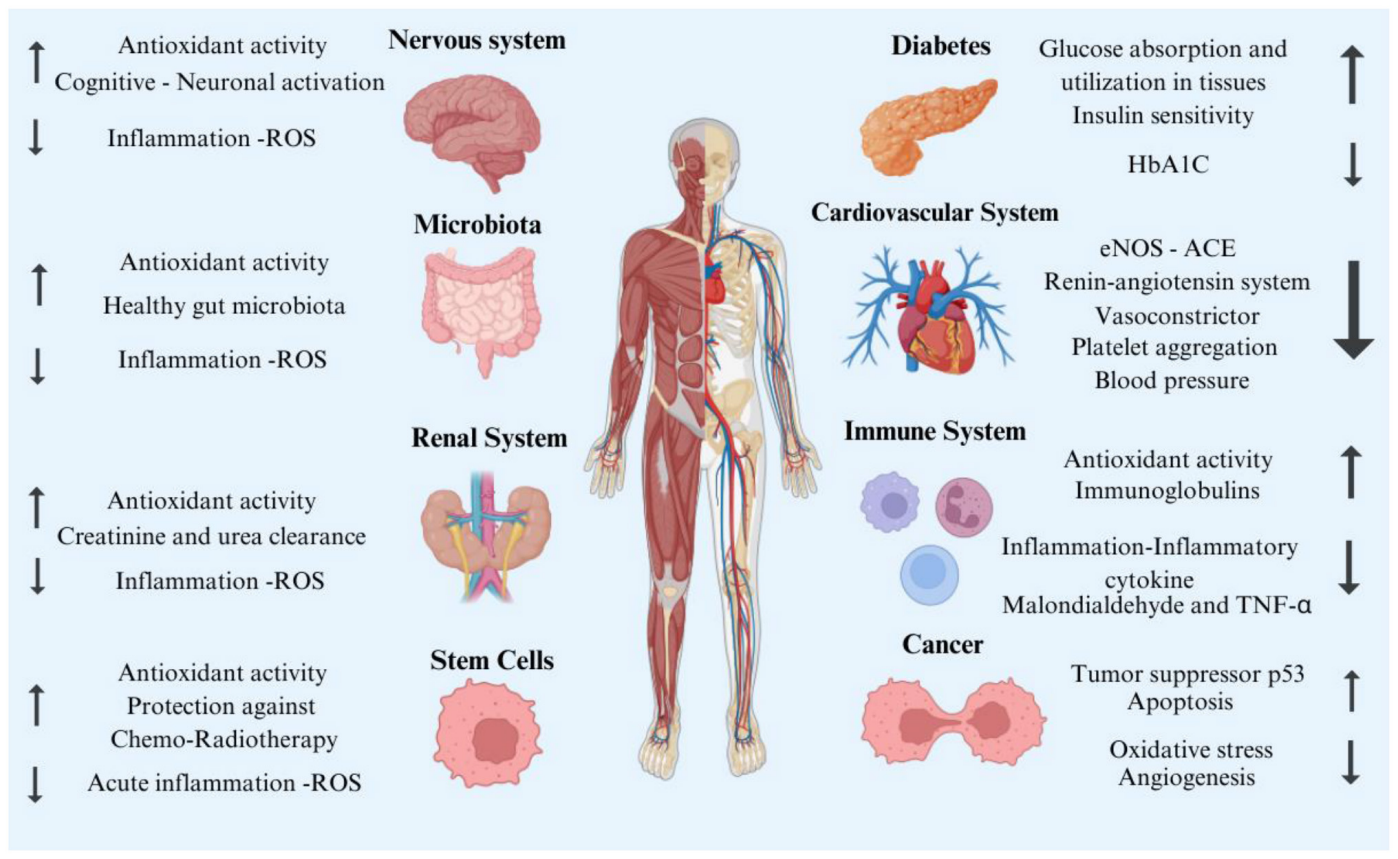
Therapeutic effects of Spirulina on human health. ROS, Reactive oxygen species; HbA1C, 3-month blood sugar average; ACE, Angiotensin Converting Enzyme; eNOS, Endothelial nitric oxide synthase; TNF-α, Tumor necrosis factor (2, 144). This diagram summarizes the systemic biological effects of spirulina on major organ systems. These effects primarily occur via oxidative and inflammatory mechanisms, such as reducing ROS and modulating inflammatory mediators. Spirulina is associated with improved neuronal function, modulation of gut microbiota composition, and increased renal clearance capacity. In metabolic regulation, it contributes to improved glucose utilization, increased insulin sensitivity, and decreased HbA1c levels. Cardiovascular effects include regulation of the renin-angiotensin system, reduction of vasoconstriction and platelet aggregation, and improved blood pressure control. The figure also highlights potential anticancer mechanisms, including immunomodulatory effects such as increased antioxidant activity and decreased pro-inflammatory cytokines, as well as apoptosis induction and suppression of oxidative stress and angiogenesis. Overall, the diagram presents the pleiotropic regulatory effects of Spirulina at the systemic level.

Due to its rich nutritional composition and its anti-inflammatory, antioxidant, and immunomodulatory properties, Spirulina has become a dietary supplement of considerable popularity on a global scale (50). This microalga exerts beneficial effects on several physiological systems, including the nervous, immune, and cardiovascular systems, and shows promising potential in the prevention and management of non-communicable diseases such as diabetes, obesity, hypertension, and cancer (50, 51). Figure 1 shows the schematic of Spirulina's therapeutic activities for human health.

### 3.1 The role of Spirulina in cancer treatment

Cancer represents a major public health concern and ranks as the second leading cause of death among non-communicable diseases worldwide. At present, cancer management primarily involves treatment approaches such as surgical intervention, chemotherapy, and radiotherapy (52, 53). In addition to the methods used in cancer treatment, nutritional interventions have also become an increasingly popular topic in recent years, and it is suggested that components considered natural antioxidants may potentially contribute to the treatment process without causing side effects (54, 55). In this context, Spirulina, which is accepted as safe by the US Food and Drug Administration (FDA) and reported to have anticancer activity thanks to the proteins, polysaccharides, polyunsaturated fatty acids, carotenoids, and phycocyanin in its structure, stands out as a remarkable bioactive ingredient (51, 56).

A study using a mouse model evaluated the effects of spirulina-derived polysaccharides on lung cancer progression. The findings showed that these polysaccharides significantly suppressed tumor development. While a marked increase in tumor volume was observed in the control group by day 15, no significant tumor enlargement was detected in the group treated with Spirulina polysaccharides (57). Similarly, Tajvidi et al. (58) showed that Spirulina exhibited anticancer activity by inhibiting free radicals, and this effect was positively correlated with Spirulina concentration (58).

In the hepatocellular carcinoma (HCC) model, Spirulina has been observed to increase survival and significantly reduce AFP levels, a tumor marker, as well as the number and volume of hepatic nodules. In addition, it has been reported that Spirulina prevents lipid peroxidation, prevents liver enzymes from leaking into the bloodstream, and increases antioxidant capacity against oxidative stress (59). Abuzed et al. (54) support the anticancer activity of Spirulina in the treatment of HCC through its regulatory effects on proinflammatory cytokines, HSPgp96, and GP3 genes (54).

In another cancer study examining the use of Spirulina, it was shown that Spirulina improved breast cancer biomarkers by inhibiting p-Akt/mTOR pathways and increased apoptotic biomarkers in cancer cells (60). Similarly, Hamdy et al. (61) reported that Spirulina significantly reduced tumor volume and metastatic burden and prolonged survival. In conclusion, Spirulina supplementation may be a promising approach for preventing tumor growth and regulating programmed cell death. A review of the studies reveals that most are based on animal models. This limits the generalizability of the findings to humans. Moreover, variations in formulation, dosage, and treatment duration contribute to inconsistent findings. Therefore, well-designed and standardized clinical studies in humans are essential to clarify the underlying mechanisms involved in cancer-related effects.

### 3.2 The role of Spirulina in diabetes treatment

Diabetes mellitus (DM) is a chronic metabolic disorder characterized by high blood sugar levels and associated with severe complications, including cardiovascular disease and chronic kidney failure (62). It is predicted that 693 million adults will be living with diabetes by 2045 (63). Although traditional antidiabetic drugs provide symptomatic improvement, their long-term use has limited effectiveness. For this reason, interest in herbal resources as an alternative and complementary approach to managing diabetes has increased (64). In these conditions, Spirulina is a promising ingredient in the treatment of diabetes with its rich nutrient profile and bioactive components (65).

Recent studies suggest that Spirulina may offer benefits for diabetes management. For example, the results of an 8-week intervention study on diabetic patients provide essential findings. Although insulin administration lowers blood glucose in advanced diabetes, chronic hyperinsulinemia in early and pre-diabetic stages contributes to insulin resistance. Tzherefore, a reduction in circulating insulin may reflect improved insulin sensitivity rather than impaired insulin production. In this context, after 8 weeks of Spirulina supplementation, diabetic participants showed significant reductions in fasting glucose, insulin, total cholesterol, LDL cholesterol, and triglyceride levels, along with a modest increase in HDL cholesterol. The concurrent decrease in glucose and insulin levels suggests improved glycemic control and enhanced insulin sensitivity. In contrast, the control group, which did not receive any additional intervention, exhibited unfavorable changes in these metabolic parameters (66). These findings are similar to another study conducted on Type 2 DM patients receiving metformin treatment, which supports the positive effects of Spirulina on glycemic control (67). At the same time, these studies show that Spirulina also improves the lipid profile in terms of cardiovascular risk factors (66, 67).

**Figure 2:**
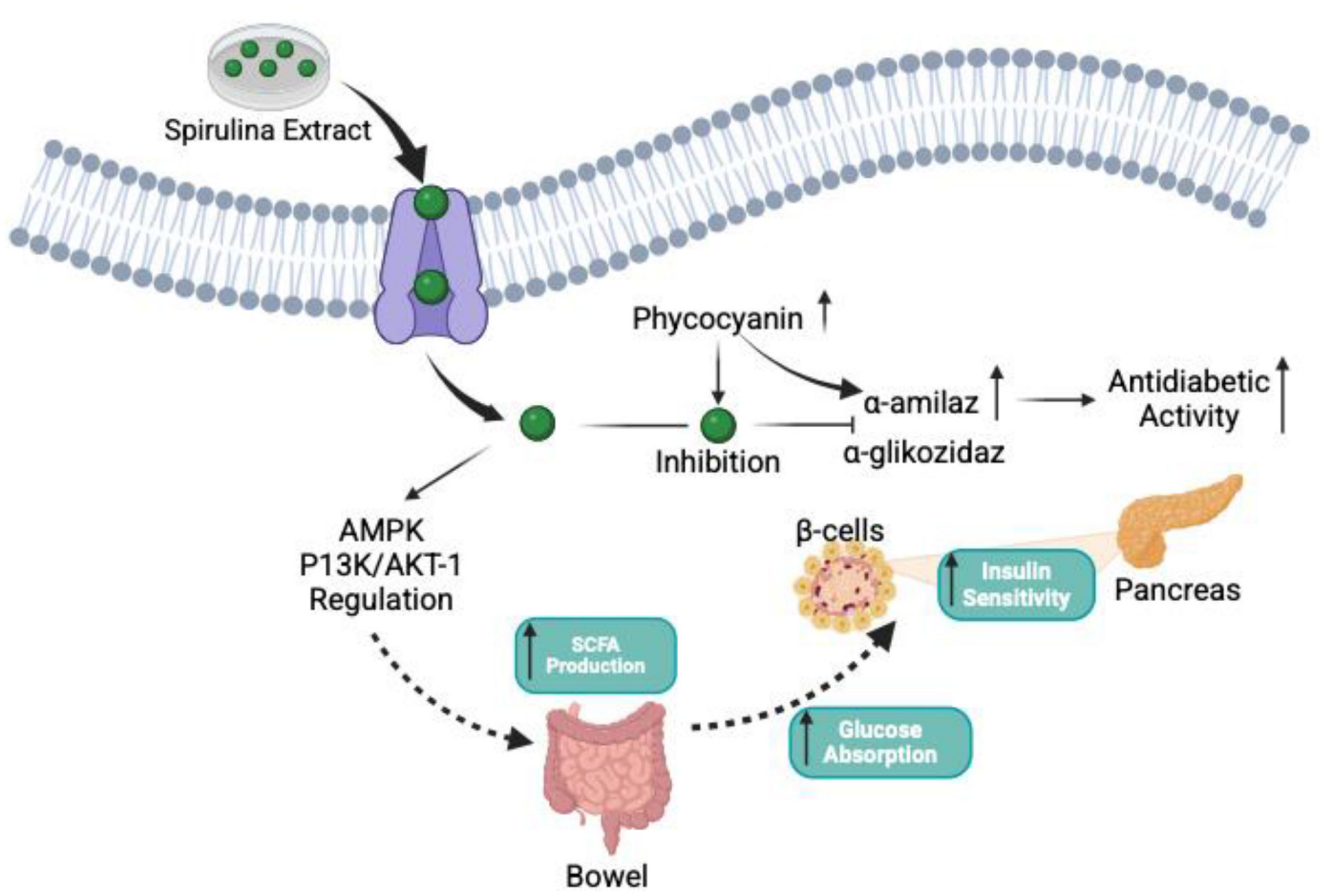
Antidiabetic mechanism of action of Spirulina. AMPK, Activating protein kinase; PI3K/AKT-1, Signaling pathway; SCFA, Short chain fatty acids (2). This diagram summarizes the proposed antidiabetic mechanisms of Spirulina. Its bioactive compounds, particularly phycocyanin, regulate key metabolic pathways, including AMPK and PI3K/AKT-1. It also improves glucose regulation by inhibiting α-amylase and α-glucosidase. The diagram further shows that Spirulina increases SCFA production in the gut, supporting β-cell function, insulin sensitivity, and overall glycemic control. Overall, the diagram integrates the potential gut and cellular mechanisms underlying Spirulina's glucose-lowering potential.

The antidiabetic activity of Spirulina is associated with activation of the insulin signaling pathway, regulation of the intestinal microbiota, and anti-inflammatory mechanisms. At this stage, *in vitro* evidence suggests that phycocyanin present in Spirulina may contribute to glycemic control by inhibiting α-amylase and α-glucosidase activities. It is emphasized that this inhibition enhances antidiabetic activity by increasing α-amylase and phycocyanin concentrations (68). Additionally, Spirulina contributes to diabetes management by modulating key signaling pathways, such as AMPK and PI3K/AKT-1, promoting gut microbiota balance through SCFA production, and enhancing insulin sensitivity by facilitating glucose uptake and utilization (68, 69). These findings support Spirulina's antidiabetic mechanism of action as a complementary treatment for diabetes. However, while some studies reported that Spirulina showed a synergistic effect by better controlling blood sugar levels in diabetic patients treated with metformin, another study reported that Spirulina supplementation did not produce a clinically significant change in blood sugar levels in individuals with newly diagnosed type 2 diabetes. These results indicate that the antidiabetic effects of Spirulina may vary depending on factors such as patient characteristics, treatment duration, and administered dose (66, 67). Studies examining the effects of Spirulina on glycemic control reveal mixed results. Although various reports have suggested that Spirulina can have a helpful impact on glycemic regulation, other studies have not identified any significant improvements. Long-term, controlled human studies are needed, as sample size, treatment duration, and the form of Spirulina used may influence the results. The potential antidiabetic mechanism of action of Spirulina is shown in Figure 2.

In addition to the mechanisms described for Spirulina, bioactive peptides derived from red seaweeds such as *Pyropia vietnamensis* exhibit potent dipeptidyl peptidase-IV (DPP-IV) and angiotensin-converting enzyme (ACE) inhibitory activities, which contribute directly to glycemic control and cardiovascular regulation. While Spirulina primarily exerts antidiabetic effects through anti-inflammatory pathways, modulation of insulin signaling, and inhibition of α-amylase and α-glucosidase, red alg-derived peptides provide complementary enzymatic targets, enhancing incretin (GLP-1 and GIP) hormone activity, and vasodilation (70). This comparison highlights that although Spirulina is a valuable functional ingredient for managing diabetes, incorporating or studying red seaweed peptides may offer additional therapeutic potential, suggesting a promising avenue for combinatorial or synergistic approaches in future research.

### 3.3 The role of Spirulina in cardiovascular diseases

Cardiovascular diseases (CVDs) are leading causes of morbidity and mortality worldwide, representing a significant public health and economic burden (71). Modifiable risk factors, such as being overweight, having disrupted glucose regulation, hypertension, and dyslipidaemia, are significant contributors to the beginning and development of CVDs (72). In recent years, nutraceuticals have attracted considerable attention due to their potential biological properties that may complement pharmacological strategies in the prevention and management of CVDs (73). In this context, Spirulina stands out as an important candidate in the treatment of CVD with its low toxicity profile and therapeutic properties (74).

Preclinical studies demonstrate Spirulina's vascular function-improving effects. Studies in aged mice have shown that Spirulina extract increases nitric oxide (NO)-mediated vasodilation in arterial smooth muscle cells. This effect has been linked to enhanced NO bioavailability and the upregulation of p-eNOS and HO-1 expression. Furthermore, Spirulina has shown promise in reducing inflammation and promoting acetylcholine-mediated vasodilation by activating NO, CO, and KATP channels (75). Similarly, in a hypertensive mouse model, Spirulina has been reported to improve vascular reactivity and positively regulate the decrease in vasodilator response caused by endothelial dysfunction. However, it was observed that this effect was not evident in mice with healthy endothelial surfaces (76).

High blood pressure and impaired lipid profile are important risk factors for CVD. Clinical research has examined the effects of spirulina on these parameters and produced positive results. It has been reported that an 8-week Spirulina-containing dietary intervention in hypertensive individuals significantly reduced systolic and diastolic blood pressure and triglyceride levels. On the other hand, changes in total cholesterol, LDL-cholesterol, and HDL-cholesterol levels were not statistically significant (77). In a study conducted in hyperlipidemic rats, Spirulina supplementation significantly reduced triglyceride, total cholesterol, and LDL-cholesterol levels, while significantly increasing HDL-cholesterol levels. The strongest effects were observed with increasing doses of Spirulina (78). Additionally, a meta-analysis reveals that Spirulina significantly reduces total cholesterol, LDL-cholesterol, and triglyceride levels while increasing HDL-cholesterol levels. These findings highlight that Spirulina supplementation may have functional effects for the prevention of CVD through its antioxidant activity (79). However, existing findings are contradictory due to methodological differences, short intervention periods, and limited sample sizes across studies. For example, Ghaem Far et al. (77) reported that Spirulina supplementation significantly reduced systolic and diastolic blood pressure in hypertensive individuals, whereas effects on the lipid profile were not statistically significant (77). Similarly, although some animal studies have shown that Spirulina lowers total cholesterol and LDL levels and increases HDL levels, the long-term implications of these effects in humans remain unclear (78). Therefore, long-term randomized controlled trials with well-defined clinical populations are needed to confirm the effects of Spirulina on cardiovascular mechanisms. Furthermore, the mechanisms by which specific bioactive compounds in Spirulina (e.g., γ-linolenic acid and phycocyanin) affect endothelial function, inflammation, and lipid metabolism require detailed investigation.

### 3.4 The role of Spirulina in the immune system

The immune system has a complex structure consisting of innate and adaptive immune mechanisms to protect the body against microorganisms and harmful pathogens (80). Immune function is negatively impacted by obesity, malnutrition, and oxidative stress, which also increases susceptibility to inflammatory diseases (81, 82). In this regard, Spirulina attracts attention due to its content of bioactive components, such as essential amino acids, phycocyanin, polysaccharides, and carotenoids, which support and enhance immune cell functions (83).

Clinical studies have shown that using Spirulina as a feed supplement improves antioxidant biomarkers, including inflammatory and anti-inflammatory cytokines, and enhances growth performance, intestinal health, and immunity in fish and poultry. In addition, although the mechanisms by which Spirulina acts have not yet been fully elucidated, these effects may be due to its bioactive components (84, 85). Table 4 shows the effects of Spirulina as a feed additive on the immune system.

**Table 4:** The role of Spirulina in feed additives on the immune system.

Working group	Effects of Spirulina as a feed additive on the immune system	References
Finned fish species	Cytokine production is increased Increased resistance to oxidative stress Mucosal immunity is increased	(85)
Nile tilapia	Antioxidant capacity has increased Increased immune response Lysozyme and phagocytic activity increased	(145)
Catfish	As a result of the addition of 7.5%−10% Spirulina; Lysozyme activity increased The immunological response is increased	(107)
Asian sea bass	As a result of the use of 2.5% Spirulina + Combined microalgae; Lysosome activity increased Total immunoglobulin (Ig) levels are increased Expression of TNF-α, IL-1β, and IGF-1 genes increased Increased immune response	(146)
Chick	Catalase activity increased Total blood antioxidant capacity increased Jejunal superoxide dismutase and glutathione peroxidase levels are increased	(84)
Chick	Combined application of Spirulina + *Dunaliella Salina*; The immune response improved with an increase in IgM and IgG values Catalase and glutathione enzymes, which determine antioxidant levels, have improved	(108)
Nile tilapia fry	Redox status and performance are increased Antioxidant capacity and immune response are increased	(147)
Chick	Effect of Spirulina on chickens given Aflatoxin B1; SOD, glutathione peroxidase, and catalase are increased Total antioxidant levels and phagocytic activity increased	(148)
Chick	Effect of Spirulina on chickens given ochratoxin A; SOD, catalase, glutathione peroxidase, total antioxidant, and immune capacity increased	(149)
Chick	IgM and IgG levels and immunological response are increased TNF-α gene expression was promoted	(150)

TNF-α, Tumor necrosis factor; IgM, Immunoglobulin M; IgG, Immunoglobulin G; SOD, Superoxide dismutase; IL-1β, Interleukin-1 beta; IGF-1, Insulin-like growth factor-1.

Much of the research in recent years has focused on animal models and *in vitro* studies, and data on their effects on the human immune system are limited. Among these limited studies, a study of individuals infected with human immunodeficiency virus (HIV) reported that Spirulina consumption significantly improved leukocyte levels, CD4 and CD3 T-cell counts, and natural killer (NK) cell counts. Spirulina has been shown to cause positive changes in immunological and clinical parameters in HIV-infected individuals (86). Similarly, a meta-analysis of systematic reviews conducted among individuals living with HIV and the hepatitis C virus (HCV) revealed that Spirulina supplements containing phycocyanin increased CD4 T cell counts and reduced viral load (87). On the other hand, intense and prolonged exercise may reduce the levels of immune cells. In a study evaluating this condition, Spirulina supplementation in young athletes resulted in significant increases in leukocyte, monocyte, eosinophil, and basophil counts (88).

Studies investigating the impact of spirulina on the immunological response in experimental animals have reported a substantial reduction in interferon-γ (IFN-γ) and tumor necrosis factor-α (TNF-α) levels in mice treated with phycocyanin. At the same time, an increase in inflammatory cytokine levels, such as IL-10, was observed in a concentration-dependent manner. Spirulina was found to support antioxidant capacity by increasing superoxide dismutase (SOD) and catalase activities (89). Conversely, Spirulina polysaccharides have been shown to enhance immune function in mice treated with cyclophosphamide (Cy), a chemotherapeutic agent. This effect is associated with the activation of chemoprotective mechanisms, which involves increasing white blood cell and lymphocyte levels, as well as restoring TNF-α, INF-γ, and IL-10 levels. Therefore, the potential of Spirulina to alleviate immunosuppression and protect immune functions is an essential basis for its evaluation as a complementary therapeutic agent (90). Recent studies have highlighted the importance of Spirulina in the immune system, mainly when used as animal feed. However, more studies are needed to reach definitive conclusions about the human immune system. The immune-system-regulating effects of Spirulina are supported by numerous experimental and clinical studies (89, 90). Current findings, though not yet extensive, suggest that Spirulina may regulate immune function in both preclinical settings and clinical contexts. A deeper understanding of the signaling cascades and cellular interactions driven by its key bioactive compounds, including phycocyanin and specific polysaccharide fractions, is essential to better establish its prospective clinical utility.

### 3.5 The role of Spirulina in weight control

Spirulina has shown positive effects on various physiological systems and may also play a role in obesity management. Obesity is considered a major contributor to various chronic conditions, largely because of excess fat accumulation and the release of certain adipokines (91). In this context, Spirulina supplementation, together with exercise, may help control obesity (92).

Spirulina may influence obesity through multiple pathways. These include limiting the infiltration of immune cells into fat tissue, preventing the build-up of fat in the liver, reducing inflammation, and promoting insulin signaling and appetite regulation (93). A clinical study of obese adults taking 6 grams of Spirulina daily for 12 weeks observed a notable decrease in body weight and body mass index, particularly when combined with exercise. In addition, it is thought that the decrease in adipokine and cytokine secretion may regulate energy metabolism by increasing insulin sensitivity (91). Similarly, another study of obese individuals who exercised reported decreases in body fat mass and increases in lean body mass. There was also a decrease in calorie intake and appetite levels. However, significant results were not always achieved in terms of all measurement parameters in individuals who only took Spirulina supplements or only exercised (94). Additionally, a meta-analysis study highlights that Spirulina supplementation can reduce triglyceride and total cholesterol levels and, therefore, may be considered as a supportive treatment for patients with Type 2 DM, metabolic syndrome, overweight, or obesity (95). Spirulina's potential effects on body weight management have shown promising results in both animal models and human studies. However, as with other health studies, a more extensive application period and a larger sample size are needed to improve our understanding of the mechanisms. Current evidence suggests that Spirulina may play a supportive role in metabolic health and body composition and therefore merits consideration as a functional food ingredient.

### 3.6 The role of Spirulina in brain health

Spirulina contains numerous nutrients and bioactive compounds that are beneficial for neural health. Thanks to its potential antioxidant, inflammatory, and neuroprotective effects, it could slow the development of neurological diseases such as Alzheimer's, Parkinson's, and multiple sclerosis. It is also reported to play an essential role in reducing damage to the cerebellum, hippocampus, and spinal cord and providing protective effects against stress (50, 96, 97).

Although the exact pathophysiology of Alzheimer's disease has not yet been fully elucidated, it is known that oxidative stress associated with the accumulation of amyloid plaques and reactive oxygen species triggers neuronal damage (98). In a study, Alzheimer's patients who received a daily 1 g Spirulina supplement for 12 weeks were assessed using the Mini-Mental State Examination (MMSE), and the results showed a significant increase in total antioxidant capacity compared to the placebo group (99). Moreover, the gut-brain axis is considered an important regulator of cognitive functions. In a study using an Alzheimer's mouse model, varying doses of Spirulina were administered over 16 weeks. The results indicated that Spirulina mitigated cognitive impairments induced by a high-fat diet, reduced β-amyloid accumulation, suppressed neuroinflammation, and prevented gut microbiota imbalance. These outcomes suggest that Spirulina may enhance cognitive performance through modulation of the gut-brain axis (100).

The neuroprotective effects of Spirulina are not limited to neurodegenerative diseases. Spirulina supplementation in rats exposed to ionizing radiation has been reported to reduce oxidative stress, brain inflammation, and pathological damage to brain tissue by regulating neurotransmitter release (101). Similarly, it is suggested that Spirulina may prevent neurodegenerative changes caused by some drugs used to treat cancer and rheumatoid arthritis and may play a role in alleviating cerebellar damage in this process. In animal models, Spirulina is predicted to reduce drug-induced neural damage and may offer a potential adjuvant therapy for patients undergoing chemotherapy (102). In addition, Spirulina is reported to reduce neuroinflammation, prevent the onset of mental disorders, and improve glial cell activation in the brain parenchyma in conditions such as mood disorders, stress, and behavioral changes, as well as in diseases such as fibromyalgia (FM), which causes extensive musculoskeletal pain (103). Spirulina has particularly noteworthy benefits for brain health, including reducing oxidative stress, suppressing neuroinflammation, and supporting cognitive function. Recent research indicates that Spirulina supplementation might offer neuroprotective benefits and enhance cognitive performance in neurodegenerative disorders, including Alzheimer's and Parkinson's disease (99, 100). However, as with the other health effects of Spirulina, most research is based on short-term studies involving small numbers of animals. Therefore, multicentre, larger-scale, and prolonged studies are needed to examine the neuroprotective mechanisms of Spirulina in greater detail.

### 3.7 The role of Spirulina in gut health

Changes in intestinal microbiota and disruptions in gene expression and metabolic enzyme activity appear to negatively affect intestinal functions (104). Spirulina contains various bioactive components that support gut microbiota homeostasis and improve gut functions (105). In particular, diets high in fat increase intestinal permeability and induce the release of inflammatory cytokines. In this context, a study conducted on rats fed a high-fat diet found that Spirulina supplementation was administered. Spirulina supplementation has been shown to prevent obesity, lower inflammatory cytokine levels, and maintain tight-junction protein levels in the gut lining. It also promotes a healthier balance of gut microbiota (106). In a rat model evaluating the effects of Spirulina supplementation on gut health, Spirulina administration showed dose-dependent effects. Improvements in intestinal morphology, preservation of intestinal integrity, and positive development of overall gut health were observed as Spirulina doses increased. This effect is thought to be related to the high protein content of Spirulina (104).

Spirulina, as a feed additive in animal nutrition, supports the immune systems of fish and poultry and has similar effects on the microbiota. Studies have reported that feeds supplemented with Spirulina improve intestinal barrier biomarkers in fish and chickens and increase the diversity of healthy microorganism communities (84, 107–109).

**Figure 3:**
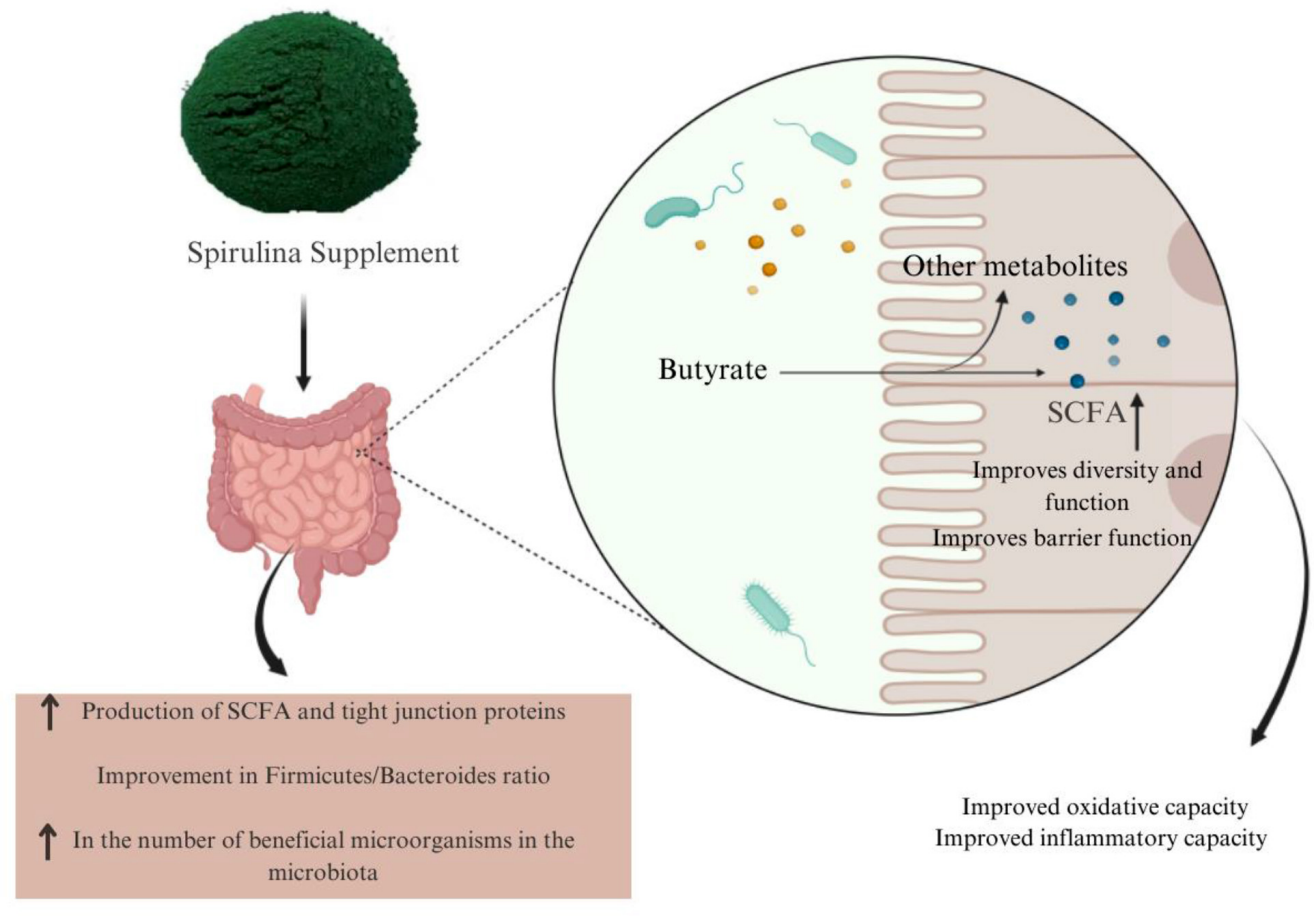
Schematic representation of the regulatory effects of Spirulina on intestinal microbiota. SCFA, Short-chain fatty acids (105). This diagram illustrates the influence of spirulina on the structure and function of the gut flora. It shows that Spirulina supplementation is associated with increased SCFA production, particularly butyrate, improved microbial diversity, and a more favorable Firmicutes/Bacteroidetes ratio. Increased SCFA production is linked to strengthened tight junctions, improved barrier integrity, reduced inflammation, and better oxidative balance. Overall, the diagram summarizes the relationship among spirulina, intestinal microbiota, and metabolite-mediated host responses.

Clinical trials have also demonstrated the benefits of Spirulina supplements for human intestinal health. For example, a survey of patients with ulcerative colitis reported that Spirulina supplementation increased total antioxidant capacity, regulated stool frequency, and improved quality of life. Spirulina has the potential to be used as an adjuvant supportive agent in the treatment of inflammatory bowel disease (110). Furthermore, in rats fed a fructose-rich diet, Spirulina supplementation increased the abundance of *Prevotellaceae* and *Lactobacillaceae* and reduced the Firmicutes-to-Bacteroidetes ratio, thereby restoring gut microbial balance. Spirulina also helped mitigate the negative impact of fructose on intestinal health by preserving microbiota diversity (111). As a result, the intestinal microbiota contributes to gut health by metabolizing Spirulina into butyrate, SCFAs, and other bioactive compounds that are readily utilized by the body (30). Figure 3 schematically shows the regulatory effects of Spirulina consumption on the gut microbiota and the potential health benefits it provides through anti-inflammatory and antioxidant mechanisms. Most evidence on Spirulina's effects comes from animal studies, making it unclear how these findings translate to humans. Factors such as diet, age, sex, and metabolic status have not been fully controlled. While preclinical studies suggest neuroprotective benefits, long-term clinical data in humans are limited, and further research is needed to determine whether these effects occur via the gut-brain axis or direct antioxidant activity.

## 4 Potential contaminants and toxic elements in Spirulina

Microalgae, particularly Spirulina, stand out as an essential dietary supplement with high nutritional value and biological benefits. While generally considered safe, medical supervision is recommended, especially for individuals with autoimmune diseases or those taking anticoagulants and hypoglycemic medications (112). Spirulina production is typically carried out in open ponds. These environments carry the risk of contamination with toxin-producing cyanobacteria and other environmental pollutants (113). Spirulina samples grown in water contaminated with heavy metals have been reported to reduce protein content and impair nutritional quality. However, the specific health risks associated with consuming Spirulina containing heavy metals have not been clearly defined (114). On the other hand, if the presence of cyanotoxin is detected in Spirulina products, gastrointestinal effects such as severe hypercalcemia, vomiting, nausea, and diarrhea have been reported (115). Therefore, the presence of cyanotoxin contamination and potential pathogenic bacteria in commercial Spirulina products highlights the need for greater attention to product safety (113).

Current studies have focused more on Spirulina's potential protective effects against neurotoxicity caused by heavy metals such as cadmium (Cd), mercury (Hg), and lead (Pb) than on its potential toxicity. Accordingly, the findings suggest that Spirulina may play a supportive role in reducing the risk of neurological damage associated with these metals (116). Similarly, Spirulina is considered a bio-healing agent that can help remove toxic metals from the body (117). However, since there is a potential for these elements to accumulate as a result of contamination during the manufacturing process, continuous monitoring of potentially toxic elements is necessary to ensure the quality and safety of Spirulina supplements (118).

On the other hand, the safety of Spirulina has also been evaluated by regulatory authorities. The United States Pharmacopeial Convention (USP) Dietary Supplements Information Expert Committee (DSI-EC) reviewed clinical, preclinical, and regulatory data, including adverse event reports, and assigned Spirulina maxima and *Spirulina platensis* a Class A safety rating, permitting the development of quality monographs in the USP–NF (119). Furthermore, while the FDA considers consumption of spirulina through various food products at 0.5–3 grams per day to be safe, its GRAS assessment also sets a possible daily upper limit of 6 grams in its accepted daily intake calculation (120). More recent research indicates that most manufacturers recommend consuming 3 to 10 grams of spirulina supplements daily, regardless of the product form (powder, capsule, or tablet) (121). However, aside from previously published safety assessments and intake estimates from independent authorities, the current literature lacks updated dosage recommendations from major regulatory bodies, highlighting a lack of consensus on standardized Spirulina intake levels.

## 5 Sustainable and technological applications of Spirulina in food systems

Population growth, changing dietary patterns, and climate change pose risks to health and sustainable food systems. Spirulina, a high-nutrient, easily produced, and environmentally friendly protein source, offers a solution to these problems and contributes to sustainable development goals by using less land (122). Its use as a biostimulant in agricultural production provides a sustainable alternative to chemical fertilizers by increasing seed germination, supporting root and shoot development, and protecting plants against abiotic stresses thanks to the amino acids, polysaccharides, and vitamins it contains (123, 124). In addition, its ability to be grown in closed photobioreactors or open systems, and its capacity to remove nitrates, phosphates, ammonium, and some heavy metals, make it possible to integrate it into environmental applications such as wastewater treatment, aquaculture, and ecosystem restoration (123, 125). The contribution of spirulina biomass to carbon sequestration and its potential use in recycling industrial food waste support sustainable food systems compatible with a circular economy approach (126).

This sustainability approach is not limited to primary production but also extends to food technologies. Alginate, derived from brown algae and certain microbial sources, stands out as an alternative packaging material to synthetic plastics due to its biodegradable structure and barrier properties against oxygen and moisture. Its three-dimensional gel structure allows for the controlled release of antioxidant and antimicrobial components, supporting the development of active packaging systems. In this context, integrating bioactives such as phycocyanin from Spirulina into the alginate matrix enables the design of both surfaces that reduce oxidative degradation and intelligent freshness indicators. This integration offers a holistic, sustainable food system approach that extends shelf life and reduces the environmental burden (127).

## 6 Spirulina in functional foods

Functional foods contain essential nutrients and bioactive compounds that promote health beyond basic nutrition. Although they resemble conventional foods, they acquire their functional properties through fortification with specific nutrients (128). Today, growing preferences for plant protein and studies on increasing food efficiency make Spirulina a critical ingredient in functional food formulations. Spirulina is attracting increasing attention in the development of functional foods because of its rich protein composition, balanced nutrient profile, bioactive compounds, and wide range of health-promoting effects (8, 129, 130).

The main producers of Spirulina are China, India, the United States, Brazil, and Egypt, highlighting the microalga's suitability for large-scale cultivation (169). Its rich nutritional profile, combined with this cultivation potential, enables its widespread use not only in the functional food industry but also in sectors such as feed, pharmaceuticals, and cosmetics (12, 17). While nutritional supplements in powder, capsule, and tablet forms remain popular particularly in the Romanian market consumer oriented products such as spirulina-enriched pasta, maki sushi, and spirulina shakes are also being developed (170, 171). Beyond food applications, Spirulina is incorporated into skincare formulations as a moisturizing, anti-wrinkle, anti-aging, and anti-acne agent (172). While current studies highlight these enriched products, there is still limited clarity regarding commercially available Spirulina products. Research focuses primarily on product enrichment rather than on existing formulations.

Considered an essential component for human health, Spirulina aims to help reduce reliance on animal products as a primary source of protein. This microalgae, which offers health-supporting effects thanks to the bioactive components it contains, comes to the fore by enriching the content of various food products such as bakery products (pasta, cookies, bread), dairy products (yogurt, ice cream, cheese), and dietary supplements (129). Studies on functional foods enriched with Spirulina are shown in Table 5. The studies summarized in Table 5 provide detailed information on the amounts and percentages of Spirulina incorporated into different food products. Wherever possible, comparative effects on nutritional composition and bioactive content relative to control samples are indicated. While many studies lack comprehensive consumer acceptance data, it has generally been observed that higher Spirulina levels tend to reduce overall acceptance, primarily due to changes in color, flavor, or aroma intensity. Information on sensory properties is limited, although some studies have reported minor effects on texture and firmness. In general, Spirulina is primarily added to foods to enrich their nutrient content, improving protein content, bioactive compounds, and functional properties, thereby supporting the development of functional foods. Moderate inclusion levels are recommended to balance nutritional benefits with sensory acceptance.

**Table 5:** Advanced functional food products with Spirulina.

Food product	Amount of Spirulina added	Enriched product features	References
Gluten-free pasta	2 different types of Spirulina were added with 1%−2%−3% ratios 1. Variety: Powdered Spirulina 2. Variety: Spray-dried commercial Spirulina	Pasta enriched with Spirulina has higher cooking quality regardless of the amount and type used. In both varieties, the color turned green after cooking. The increase in Spirulina content improved the pasta's chemical composition and antioxidant capacity.	(151)
Pasta	2% to 5% CCC 540 Spirulina powder was used	Green pasta enriched with Spirulina significantly increased the protein, total phenol, flavonoid, iron, and calcium content, and GLA and docosahexaenoic acid levels, with GLA and docosahexaenoic acid levels increasing 2–2.5 times. Increasing the concentration of Spirulina improved functional properties while maintaining cooking quality and texture integrity, increasing consumer appreciation and purchase willingness.	(133)
Wheat noodles	Spirulina powder was used at 0%, 1%, 2%, 3%, and 4%.	Noodles containing 2% Spirulina improved their physicochemical and structural properties by reducing gliadin, resulting in the most favorable chewiness, hardness, elasticity, and taste profile.	(152)
Wet noodles	Spirulina powder was used at 0%, 1%, 5%, and 10%.	According to sensory evaluation, noodles containing 1% and 5% Spirulina are acceptable in appearance and aroma. However, the best results in terms of taste, smell, aroma, and texture were obtained with noodles containing 1% Spirulina. Spirulina-enriched wet noodles offer a better nutritional profile and functional benefits.	(153)
Pasta	Spirulina powder was used at 5%, 10%, 15%, and 20%.	With the increase in Spirulina dose, total phenolic, flavonoid, and antioxidant capacity increased proportionally, and cooking quality improved. Increased antioxidant capacity positively affected kidney, liver, and hematological-biochemical parameters in mice.	(154)
Gluten-free pasta	Spirulina powder was used at 5%, 10%, and 15% concentrations.	Protein, ash, and fat contents increased significantly in gluten-free pasta containing 10% and 15% Spirulina, and high Spirulina concentration increased phenolic compounds and antioxidant capacity.	(155)
Gluten-free sourdough bread	3% encapsulated and unencapsulated Spirulina formulations	Encapsulated Spirulina increased the antioxidant capacity, phenolic compounds, and sensory acceptance of bread, while encapsulated Spirulina enriched the protein, amino acid, folate, mineral, and fiber content. With the addition of pseudocereals and psyllium, the nutritional value of bread has further improved, particularly in protein, fatty acids, essential amino acids, and soluble dietary fiber.	(156)
Bread made from 4 different wheat flours	The amount of Spirulina varies depending on the amount of flour used. 1.5% Flour: 6 g Spirulina 2.5% Flour: 10 g spirulina	Increasing the amount of Spirulina improved the bread's nutritional profile and antioxidant capacity, and it gave the bread a greener color.	(157)
Quinoa flour gluten-free biscuits	Spirulina powder was used at rates of 0%−3%−6%−9%	Increasing the Spirulina concentration increased the protein, fat, ash, and fiber content of cookies and snacks while decreasing the hardness. Cookies containing 3%−6% Spirulina were found to be more suitable in terms of sensory quality, whereas higher rates reduced satisfaction with color, taste, and odor.	(158)
Gluten free cake	Spirulina was used at 0%, 1.5%, and 3%.	Increasing the Spirulina content enhanced the total soluble polyphenol content and antioxidant activity of the cakes, tightened the structure, and caused the formation of green tones. Spirulina should be used in higher forms to better explore its nutritional and bioactive properties.	(159)
Mini cake	The amount of Spirulina varies depending on the amount of blueberries used. 4% Blueberries: 1% Spirulina 8% Blueberries: 2% Spirulina	According to texture and sensory analysis, the most suitable cake for consumption contained 1% Spirulina. However, as the Spirulina and blueberry concentration increased, the antioxidant capacity also increased.	(160)
Halloumi cheese	Spirulina powder was used at 0%−0.5%−1%−1.5% rates	The addition of Spirulina significantly affected the color, texture, and nutritional content of halloumi cheese. 0.5% Spirulina resulted in a greenish color, while 1%−1.5% ratios enhanced the mineral and nutritional profile. However, the most appropriate rate for consumption is 0.5% Spirulina addition.	(161)
Yogurt from milk with high somatic cell count	Spirulina powder was used at rates of 0.25%, 0.50%−1%	The addition of Spirulina increased the lipid and protein content of yogurt made from both milk types. Yogurt with 0.25% Spirulina improved the nutritional profile and quality while preserving sensory properties.	(162)
Yogurt from low somatic cell count milk			
Prebiotic yogurt	Spirulina powder was used at rates of 0%−0.5%– 0.75%–and 1%	As the amount of Spirulina increased, the nutritional composition, fiber content, and number of probiotic microorganisms in the yogurt increased. This shows that adding Spirulina enhances the yogurt's prebiotic properties.	(163)
Vegan basil pesto sauce	Spirulina was used at 1% and 2% levels.	As the Spirulina concentration increased, the protein, iron, calcium, polyphenol, flavonoid, and antioxidant content of the pesto sauce increased, and its color turned darker green. Both concentrations were suitable for consumption, but the highest quality parameters were observed in sauces containing 1% Spirulina.	(164)
Fermented sports drink	Spirulina powder was used at rates of 0.25%, 0.50%, and 0.75%	Fermented sports drinks containing Spirulina prepared with lemon and mint juice increase vitamin, mineral, antioxidant, and total phenolic compound content. The addition of Spirulina increased the number of probiotic bacteria and preserved the drink's probiotic properties. Significantly, 0.5% Spirulina accelerated fermentation while maintaining sensory acceptability.	(130)
Ice cream	Sample 1: 12 g Spirulina Sample 2: 24 g Spirulina	Ice creams containing 24 g of Spirulina have higher protein, calcium, iron, and vitamin B2 and A content than those containing 12 g of Spirulina. Both Spirulina levels were deemed suitable for consumption.	(165)
Probiotic ready cereal drink	Spirulina concentrations vary depending on the amount of Sorghum flour used 50% Sorghum Flour: 1% Spirulina. 48% Sorghum Flour: 3% Spirulina. 46% Sorghum Flour: 5% Spirulina.	The increased addition of Spirulina strengthened the product's nutritional profile, but the reduced amount of flour made it difficult to consume. The crispest and shiniest structure was observed at the 50:1 cereal-to-drink ratio, which was found to be most suitable for consumption. Additionally, the addition of Spirulina increased the viability of probiotic microorganisms.	(166)
Vegan biscuits	Sample set 1 Control group: No Spirulina biomass wheat flour substitute SB5%: 5% unencapsulated Spirulina biomass SB10%: 10% unencapsulated Spirulina biomass SB15%: 15% unencapsulated Spirulina biomass	Sensory analyses indicated that adding 15% or more of non-microencapsulated Spirulina biomass to cookies negatively impacted the product's sensory properties. There was an 18% decrease in consumer acceptance and a 24% decrease in purchase intention. The nutritional profile was not evaluated for the first sample set.	(20)
	Sample set 2 Control group: No Spirulina biomass SB20%: Wheat flour + 20% unencapsulated Spirulina biomass MSB20%: Microencapsulated Spirulina biomass	The addition of 20% Spirulina, used as a wheat-flour substitute via microencapsulation, did not negatively affect sensory acceptance. Protein content increased by approximately 40%, while iron content increased by an average of 54%. It is believed that biscuits produced in this way can be labeled as an “iron source.”	
Pasta	Spirulina was added in specific proportions to fresh pasta made with semolina flour (*Triticum*). Four pasta samples were obtained. According to	Spirulina supplementation resulted in a greener color in the pasta. Specimen P7, in particular, received the highest sensory scores, including overall appearance, color, taste, and texture. Specimen P10, however, experienced lower palatability scores. However, sample P10 had the highest protein and dietary fiber content.	(135)
	these samples: P3: 3% Spirulina added P5: 5% Spirulina added P7: 7% Spirulina added P10: 10% Spirulina added		
Pasta	Amaranth flour wheat pasta (AWP), made from a blend of wheat flour and amaranth flour, is enriched with specific Spirulina (SPP) ratios. These ratios are: 1. 38% wheat flour + 38% amaranth flour: 6% SPP 2. 37% wheat flour + 37% amaranth flour: 8% SPP 3. 36% wheat flour + 36% amaranth flour: 10% SPP	Pasta enriched with amaranth and SPP exhibited good appearance, texture, and green color, along with high nutritional value. Protein content was 24.60% in samples with 10% SPP, while protein content was 15.26% in samples without SPP. Furthermore, total essential amino acid content increased in the 10% SPP group, improving the overall nutritional quality of the product	(32)
Mayonnaise	When creating mayonnaise prototypes, 0.5% SPP was added.	The natural pigments found in SPP imparted a distinct green color to the product. The resulting product exhibited high emulsion stability due to SPP, demonstrating its potential as a stabilizing agent.	
Yogurt	*Spirulina platensis* commercial grups: 0,5%−1.0 %– 1.5%−2.0% *Spirulina platensis* agglomerated with maltodextrin 30% grups: 0.5%– 1.0 %−1.5%−2.0%	Yogurt enriched with clumped *Spirulina platensis* exhibited a favorable fermentation profile, high viability of probiotic bacteria, and high nutritional value. Protein content increased to 5.63% in samples containing 2.0% *Spirulina p*., while the control samples without Spirulina p. had a protein content of 3.96%. Furthermore, the inclusion of bioactive compounds such as phycocyanin, β-carotene, and chlorophyll a significantly improved the product's antioxidant capacity and overall functional quality.	(167)
Potato snacks	*Spirulina platensis* powder was used at rates of 1.5%−2.5%−3.5%−5.0%	Adding *Spirulina platensis* at concentrations of 1.5–5.0% to potato snacks significantly increased their protein, fiber, mineral, and antioxidant content, enhancing their functional properties. The addition of Spirulina also resulted in a crispier texture and a darker, greenish color. Sensory evaluations showed that a 3.5% Spirulina concentration provided the most balanced and consumer-acceptable level of flavor and nutritional enrichment.	(142)
Jackfruit-based meat analog formulations	Spirulina powder was used at: F1: 0.91% F2: 0.92% F3: 0.95% F4: 0.87%	Adding less than 1% Spirulina to a jackfruit-based meat analog increased the product's protein and iron content and maintained flavor stability during storage, thanks to its antioxidant effects. Despite the low Spirulina content, a meat-like, fibrous structure and a safe shelf life of 60 days yielded a nutritious and sustainable meat alternative.	(168)

CCC, Spirulina strain from the CCC collection; AWP, Amaranth flour wheat pasta; SPP, Spirulina platensis protein; P3, P5, P7, P10, pasta samples where semolina flour was replaced with spirulina powder at 3%, 5%, 7%, and 10%; SB, Spirulina biomass; MSB, Microencapsulated Spirulina biomass.

Effects compared with control, sensory observations, and consumer acceptance information are included in tables where possible and discussed in the main text.

The integration of Spirulina biomass into functional foods has significant potential thanks to the high-quality proteins, minerals such as iron and calcium, and various bioactive compounds it contains (20). As shown in Table 5, incorporating Spirulina into various food products, such as baked goods, dairy items, and beverages, can improve their nutritional value. Increases in protein, essential amino acids, pigments, and antioxidant compounds have generally been reported in these products. However, current findings are limited mainly to chemical compositional analyses, and practical parameters such as sensory acceptance, consumer satisfaction, and product stability are not adequately addressed. In this context, low awareness of Spirulina's benefits is a key factor limiting market growth. While sensory attributes such as taste, odor, color, and aroma hinder consumer acceptance, new technologies such as microencapsulation, fermentation, and enzymatic processing offer promising results in mitigating these disadvantages (20, 131). The fact that existing studies are generally conducted for short periods, with small samples, and based on self-reporting limits the generalizability of the results. Therefore, a comparative analysis of the acceptance level of Spirulina in different cultural and regional markets in future research will enable a more comprehensive assessment of its potential (132). Furthermore, future studies should prioritize establishing standardized evaluation protocols to improve the sensory properties of spirulina, as well as investigating the impact of innovative culinary and technological processes, such as flavor masking strategies, food pairings, and combining with other ingredients.

## 7 Conclusion

This review underscores the substantial potential of Spirulina (*Arthrospira platensis*) as a sustainable and functional food ingredient, due to its remarkable nutritional composition and rich array of bioactive compounds. Spirulina is abundant in high-quality proteins, essential amino acids, complex carbohydrates, polyunsaturated fatty acids, vitamins, minerals, and phenolic compounds, which collectively support multiple health-promoting functions. Preclinical and limited clinical evidence indicate that Spirulina exhibits antioxidant, anti-inflammatory, antimicrobial, and anticancer properties, contributing to beneficial effects on conditions such as diabetes, obesity, cardiovascular diseases, cancer, and gut-related disorders. These findings highlight Spirulina as a promising candidate for incorporation into health-enhancing foods, nutraceuticals, and functional products. Its low land and water requirements, high biomass yield, and rich bioactive profile also position it as a sustainable protein source for future food systems. However, variations arising from production conditions and potential contamination with heavy metals or other toxic elements underscore the need for rigorous quality control and standardized cultivation and extraction practices. Furthermore, improving sensory attributes and optimizing formulation strategies are essential to enhance consumer acceptance and facilitate broader application in diverse food matrices. In the long term, comprehensive studies are also needed to unravel the molecular mechanisms by which Spirulina and its bioactive constituents influence metabolic and immune pathways. Addressing these research areas will help establish Spirulina as a validated, functional, and accessible resource to advance human health and food science innovation.

Despite the promising findings summarized in this review, several limitations should be acknowledged. The majority of available evidence is derived from preclinical studies, while well-designed, long-term randomized clinical trials remain limited. Variability in cultivation conditions, extraction methods, and dosage protocols across studies also complicates direct comparison of results and standardization of health claims. Furthermore, bioavailability, optimal intake levels, and long-term safety profiles of Spirulina and its specific bioactive compounds require further clarification. Future research should therefore prioritize standardized methodologies, large-scale human intervention studies, mechanistic investigations at the molecular level, and comprehensive safety assessments to enhance the translational applicability of Spirulina in functional food systems.
